# The Wheel of p53 Helps to Drive the Immune System

**DOI:** 10.3390/ijms24087645

**Published:** 2023-04-21

**Authors:** Barbara Łasut-Szyszka, Marek Rusin

**Affiliations:** Center for Translational Research and Molecular Biology of Cancer, Maria Skłodowska-Curie National Research Institute of Oncology, Gliwice Branch, 44-101 Gliwice, Poland; barbara.lasut-szyszka@io.gliwice.pl

**Keywords:** p53, immunity, transcriptome, virus, negative-feedback loops

## Abstract

The p53 tumor suppressor protein is best known as an inhibitor of the cell cycle and an inducer of apoptosis. Unexpectedly, these functions of p53 are not required for its tumor suppressive activity in animal models. High-throughput transcriptomic investigations as well as individual studies have demonstrated that p53 stimulates expression of many genes involved in immunity. Probably to interfere with its immunostimulatory role, many viruses code for proteins that inactivate p53. Judging by the activities of immunity-related p53-regulated genes it can be concluded that p53 is involved in detection of danger signals, inflammasome formation and activation, antigen presentation, activation of natural killer cells and other effectors of immunity, stimulation of interferon production, direct inhibition of virus replication, secretion of extracellular signaling molecules, production of antibacterial proteins, negative feedback loops in immunity-related signaling pathways, and immunologic tolerance. Many of these p53 functions have barely been studied and require further, more detailed investigations. Some of them appear to be cell-type specific. The results of transcriptomic studies have generated many new hypotheses on the mechanisms utilized by p53 to impact on the immune system. In the future, these mechanisms may be harnessed to fight cancer and infectious diseases.

## 1. Introduction

The p53 tumor suppressor protein is best known as an inhibitor of cell cycle progression and an inducer of apoptosis. Paradoxically, its ability thereof to stimulate expression of cell cycle inhibitors or the positive regulators of apoptosis is not required for its tumor suppressive function (reviewed by Aubrey et al. [1]). Thus, we are far from fully understanding its biological activities and, as always, the p53 protein maintains its power to surprise us. One of its lesser known and less studied functions is the capability to regulate the immune system. A tumor suppressor is expected to stimulate immunity. Yet, p53 positively regulates expression of some immunosuppressive genes. The plausible explanations are that p53 participates in negative feedback loops in immunity-related signaling pathways or that p53 favors one type of immunity over the other. In this review we present some proven or potential immunity-related functions of p53, hoping that it will constitute an inspiration to pursue research paths that have started to appear. Various review papers on the role of p53 in immunity have been published recently [2,3,4,5] or longer ago [6]. We present this topic inspired by transcriptomic data generated by our research team [7], but primarily by others [8].

## 2. The p53 as a Transcription Regulator

**Figure 1:**
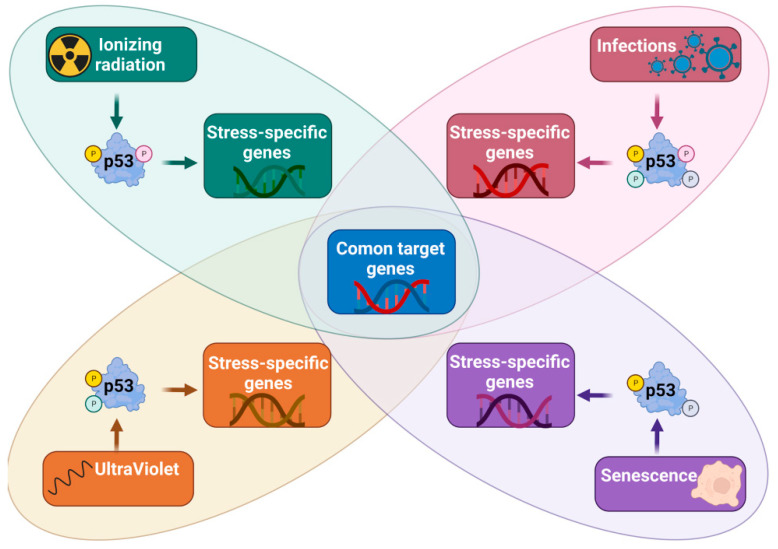
Schematic representations of various p53 forms generated by posttranslational modifications induced by stress factors encountered by cells. Activated p53 stimulates expression of a set of genes regardless of the type of stress (common target genes), whereas other genes (stress-specific genes) are preferentially activated only when p53 is modified in a particular fashion. While common target genes are well known, the stress-specific genes are not well recognized. P53 modifications induced by infections have been poorly studied. Created with BioRender.com (accessed on 14 February 2023).

The 53 protein was first identified as a cellular molecule binding to a protein coded by a tumor virus. This might already suggest that p53 plays a role in innate antiviral immunity. The major biochemical activity of p53 involves a sequence-specific binding to DNA and regulation of the transcription rate of target genes. The p53 protein consists of three major domains. The N-terminal fragment includes two transcription-activating domains (TAD). The central domain is well-structured and contains the globular fragment, which binds DNA in a sequence-specific manner. The C-terminal fragment comprises the tetramerization domain. In contrast to other transcription regulators, which bind to DNA as monomers, dimers, or heterooligomers, p53 binds to DNA as a tetramer [9]. It is reflected in the structure of the target sequence or the p53 response element (RE) consisting of two decameric half-sites RRRCWWGYYY(N_0–13_)RRRCWWGYYY (R = A/G, Y = C/T, W = A/T), which may be separated by one to thirteen nucleotides, although the REs with the strongest ability to activate genes have no intervening nucleotides. The half-sites consist of quarter sites arranged in a palindrome-like manner—RRRGW followed by WCYYY. This arrangement allows each quarter site to be bound by one p53 monomer of tetrameric p53. In some conditions, this organization enables the p53 tetramer to bind to the DNA target on condition that the sequence of one quarter fails to match the consensus sequence. These REs are called three-quarter sites [10]. The binding of p53 to the three-quarter sites is promoted when the p53 monomers strongly bind to each other. Such a strong binding of monomers occurs in the presence of some posttranslational modifications of p53. For instance, phosphorylation of serine 392 of p53 promotes tetramerization. However, in all likelihood, many more covalent modifications of p53 or protein–protein interactions also support this process [11,12,13]. Thus, the selection of a binding site critically depends on a set of covalent modifications of p53 monomers and this, in turn, is regulated by the activity of dozens of enzymes that decorate p53 with phosphorylations, acetylations, methylations, ubiquitinations, and other modifications ([14] and refs therein). The activity of enzymes that modify p53 relies on the nature of a stress factor, hence p53 modified in response to DNA double-strand breaks differs from p53 modified in response to DNA damage caused by ultraviolet (UV) radiation [15]. This stress-regulated flexibility of p53 to bind different loci enables a precise response of cells to specific damage caused, for instance, by ionizing radiation (IR, causing DNA double-strand breaks) or UV. Hence, some p53 target genes are preferentially activated by IR, others are preferentially activated by UV, whereas others respond to either of the stressors in question (Figure 1). According to this model, various stress factors activate different target genes, since they stimulate the p53 modifying enzymes, which decorate p53 in a different fashion, enabling p53 tetramers to bind REs located in various genes. A good case in point is represented by the results of our recent experiments. The p53 protein is strongly activated by the concerted action of two substances—actinomycin D and nutlin-3a (henceforth abbreviated to A+N). Actinomycin D induces nucleolar stress and activates kinases, which phosphorylate p53, whereas nutlin-3a antagonizes the major negative regulator of p53—the MDM2 protein. These two substances synergize in the phosphorylation of p53 and in the activation of some of the target genes thereof [16]. A kinase inhibitor known as CHIR-98014 modulates this activation in a gene-dependent fashion. The degree of activation of some p53-regulated genes is not changed by the inhibitor or is even slightly augmented (e.g., *FAS*, *PMAIP1*, *BBC3*, *CDKN1A*), whereas the upregulation of other genes is thereby prevented (e.g., CASP1, CRYAB, H19, STING1, TREM2) [7,17]. Thus, the logical conclusion is that a kinase inhibited by CHIR-98014 is not involved in the activation of the first group of p53 targets but is indispensable for the activation of the second group. These observations generate the hypothesis that the CHIR-sensitive kinase phosphorylates p53 in a manner that allows it to bind to the new gene-regulatory sequences or transcription factors controlling the expression of new genes.

P53 has two transcription activating domains located on the N-terminal fragment (TAD1 and TAD2). The N-terminus is extensively covalently modified in response to stress. Again, different stress factors induce different sets of modifications within TAD1 and TAD2. This, in turn, modifies the interactions of TADs with the gene regulatory proteins: general transcription factors, mediator complex, and chromatin modifying proteins [18]. It may be hypothesized that when the TADs thereof lack the modifications that allow them to interact with the proper transcription regulators, this gene will not be activated, even if p53 binds to a sequence localized in a gene promoter or enhancer. From the results of high-throughput experiments, it is well known that p53 binds more genes than it activates in a given cell type under particular stress conditions [19]. The discussion is presented to rebuke the simplistic notion that p53 is activated like a simple switch in response to a stress factor and mobilizes a fixed set of target genes leading to cell cycle arrest, apoptosis, or DNA repair. In reality, there are probably several forms of p53—molecules with the relevant sets of covalent modifications which activate various sets of target genes aimed at precise response to a given stress factor [20]. We have only started to comprehend this complicated picture. However, there are significant barriers on this path. One of these is the notion that we have already understood all important aspects of p53 functioning. This protein appears as a familiar element in respected textbooks, and researchers no longer study p53 as intensely as they did in the 1990s. An increase in the number of papers on p53 reached a plateau in the last decade. Another barrier is the lack of experimental procedures that would enable us to scrutinize the complicated interactions among p53 and its activators, other transcription regulatory proteins, chromatin modifying enzymes, chromatin itself, etc. A third barricade results from the limitations of our research models, e.g., laboratory animals are kept in sterile conditions, while those in the wild are exposed to complex mixtures of viruses, bacteria, fungi, animal parasites, etc.; these conditions seem virtually infeasible to duplicate in the laboratory. Moreover, because murine and human immune systems function in a different fashion [21], the role of p53 in immunity may be different in these two species. While we may gain some knowledge from knockout mice on the role of p53 as an antiviral protein, it is not certain that human p53 plays an identical role. Our accumulated knowledge on p53 as a tumor suppressor is enormous, while our comprehension of p53 as an immunity regulator is much poorer. Probably, the role of p53 as a regulator of immunity does not rival its role as a tumor suppressor. However, the immunity-related functions of p53 are partially associated with its anti-oncogenic functions; e.g., hypothetically, p53 may make incipient cancer cells better targets for cytotoxic lymphocytes or natural killer (NK) cells.

## 3. The Hints That p53 Is Involved in Immunity—Tumor Viruses Antagonize p53

The notion that p53 might boost immunity could have been guessed shortly after its discovery. After all, p53 was detected as a cellular protein strongly interacting with a protein (large T-antigen) of a virus named SV40. This simian vacuolating virus 40, naturally occurring in macaques, was introduced into humans via contaminated polio vaccines. Should a viral protein interact with a cellular protein, it is generally either to inactivate it (if the cellular protein interferes with the virus life cycle) or to hijack the protein to serve the needs of the virus. In the case of p53 and large T, the purpose of their interaction was not initially apparent. In principle, this interplay could enable the virus to inactivate p53 if its functioning interferes with replication of the virus. After initial controversy, it was independently demonstrated that human wild-type p53 inhibits the activities of large T-antigen [22,23]. Thus, wild-type p53 antagonizes SV40 replication. The controversy arose from the fact that a study demonstrating the inability of human p53 to inhibit the large T-antigen was performed with mutant p53, which was considered wild-type at the time of the experiments. The controversies persisted, as some observations suggested that the ability of p53 to interfere with SV40 DNA replication was due to overexpression of p53 in the experimental setting. It has been argued that once the amount of p53 is at a physiological level, it fails to influence SV40 replication [24]. Thus, the true influence of p53 on replication of the virus in question has not been firmly established. In the meantime, p53 has been found to have the ability to bind specifically to DNA [25]. This was one of the first biochemical activities of p53 to be discovered. It soon became apparent that the site-specific binding of p53 to DNA is inhibited by the large T-antigen [26]. It has also been demonstrated that the SV40 large T-antigen may inhibit the ability of p53 to act as a transcription activator [27]. Thus, it was proved that p53 has the ability to antagonize SV40 replication, and the SV40 large T-antigen may antagonize the main function of p53—the activation of gene expression. To further complicate matters, the result of SV40 infection depends on the host cells. Monkey cells are permissive to viral replication (the virus may undergo the complete replication cycle, frequently leading to cell lysis). Mouse cells are not permissive to SV40 replication—the virus may infect them but viral DNA replication is undetectable and progeny viruses are not produced. However, infection of mouse cells leads to their stable transformation with fragments of viral DNA covalently integrated into cellular DNA. Infection of human cells by SV40 leads to a different outcome. As in mouse cells, transformation may occur, which is manifested by altered cell morphology and growth properties, but as in monkey cells, virus production may also occur. This combination of features has led to the use of the term “semi-permissive” to describe human cells in this context (reviewed by Martini et al. [28]). Thus, it appears that SV40 trumps p53 in primate cells under physiological conditions. However, the question arises of whether it occurs in all cells. After all, we observe only the positive outcome (virus progeny), and we do not know how many cells the virus entered but was prevented from replicating. A recent study revealed that the struggle between p53 and the large T-antigen begins very early in infection. A combination of live cell imaging and single-cell analysis showed that a subset of cells activate p53 immediately upon virus entry. In these cells, the infection did not lead to production of large T-antigens. Hence, should a cell quickly activate p53 or should the virus enter a cell with activated p53, the virus does not replicate. In addition, artificial elevation of p53 expression reduced the efficiency of infection. Moreover, p53 interferes with virus replication by binding to viral DNA in the promoter region for the large T-antigen, preventing activation of the promoter by Sp1 transcription factor [29]. Thus, for unknown reasons, only some cells are able to rapidly elevate p53 activity following virus entry—they are lucky or resourceful enough to prevent the virus from replicating. It appears that the quick response of p53 is to prevent SV40 replication.

SV40 is not the only tumorigenic virus encoding a protein that inactivates p53. P53 antagonists are also produced by human papilloma viruses (HPVs), which frequently induce cancers in humans, and by adenovirus 2 (reviewed by Aloni-Grinstein et al. [30]). The common view is that oncogenic viruses inactivate p53, since p53, by inhibiting the cell cycle, limits the cellular resources required by viruses to replicate their genomes. However, this model may be oversimplified, as there is evidence that p53 acting as a transcription regulator activates many immunity genes, which code for the proteins restricting virus replication [31]. Therefore, not only do the viruses antagonize p53 to thrive in the replicating cells, but they also disable its ability to promote the expression of immunity genes. In the last two decades of the 20th century, the study of the virus–p53 relationship was virtually limited to SV40, HPV, and Ad2. More recent studies have demonstrated that non-oncogenic viruses also code for proteins modulating the activity of p53. The following section contains some examples.

## 4. p53 Can Be Regulated by Non-Oncogenic Viruses

Zika virus (ZIKV) is a mosquito-borne RNA virus of the *Flaviviridae* family, which is present in Latin America. It has a considerable impact on society, since the ZIKV infection in pregnant women is associated with congenital microcephaly [32]. The p53 pathway is activated in human neuronal progenitor cells infected with ZIKV, and this activation is associated with the induction of apoptosis. The activation of p53 is an early and specific event during cells’ infection with ZIKV [33]. Apparently, the p53 pathway senses the presence of the virus and responds with the activation of p53-target genes, leading to apoptosis, which destroys the host of the replicating virus. In an attempt to dissect the mechanism of p53 activation, Slomnicki et al. [34] found that Zika virus capsid protein (ZIKV-C) enters the nucleolus and induces nucleolar stress, which is known to be an activator of p53 [35]. In a recent study, Li et al. [36] demonstrated that the non-structural protein 5 (NS5) of ZIKV interacts with and stabilizes p53 and induces apoptosis of infected cells. Thus, it appears that ZIKV utilizes at least two of its proteins (capsid and NS5) in order to activate p53 and apoptosis. The capsid protein of another flavivirus that causes dengue fever also induces nucleolar stress and apoptosis [34]. However, we have only begun to comprehend the complex interaction between flaviviruses and the p53 pathway. Based on these data, we can build a model according to which the activation of p53 in at least two flaviviruses is mediated by nucleolar stress induced by a capsid protein. Activated p53 induces apoptosis, destroying the host cell. The question is whether apoptosis promotes or inhibits the spread of the virus. Is p53 an antagonist or an ally of the virus? Is the nucleolar stress a sneaky trick used by the virus to activate p53 in order to induce apoptosis? These questions have no obvious answers, and some viruses use apoptosis to spread themselves (see below). On the other hand, at least in principle, without p53 the consequences of infection could be worse.

The capsid protein is also responsible for the p53-dependent apoptosis induced by West Nile virus—another member of *Flaviviridae*. Yet, the mechanism of p53 activation is different in this scenario, since the capsid protein induces sequestration in the nucleolus of MDM2, which is the major negative regulator of p53. Without inhibition from MDM2, p53 is activated and promotes expression of the proapoptotic target genes [37].

A systematic approach toward the identification of novel modulators of p53 encoded by genomes of emerging viruses was used by Alzhanova et al. [38]. These authors synthesized, validated, and tested 172 open reading frames of unknown function from emerging viruses (e.g., SARS-CoV, ZIKV, Kaposi sarcoma-associated herpesvirus—HSHV) in a functional screening of p53 signaling. The study revealed novel mechanisms of p53 virus interactions and the binding of two viral proteins KSHV orf10 and ZIKV NS2A to p53. These findings have reinforced the notion that probably most viruses, including RNA viruses, interfere with p53 functions.

In the case of *Flaviviridae,* the accumulated knowledge on the interactions among viruses and the p53 pathway is very limited, partly because the impacts of these viruses on public health have appeared only recently (West Nile virus, Zika virus). In contrast, influenza A virus (IAV) has an enormous influence on the health and economics of populations worldwide. Furthermore, this virus has been studied for decades. Surprisingly, it has been learned that the apoptosis of infected cells is essential for efficient propagation of IAV [39,40]. As in the case of *Flaviviridae*, the infection of cells with IAV leads to apoptosis mediated by the activation of p53 [41]. IAV prevents the interaction between p53 and its negative regulator—the MDM2 protein. The amount of MDM2 is not diminished, but it no longer interacts with p53, probably due to the activity of viral nucleoprotein NP [42]. In a more recent study utilizing the A549 cell line derived from lung cancer and permissive for IAV, the authors found that p53 facilitates approximately double the propagation of the virus in cultured cells. One of the mechanisms is downregulation by p53 of the interferon-stimulated genes *IFITM1*, *IFITM2*, and *IFITM3*. The proteins that are thereby coded restrict the infectivity of diverse viral pathogens including IAV. Interestingly, the authors reported that IAV infection modulates expression of 1508 genes in wild-type cells, whereas in p53 knockouts this number is reduced to 506. Hence, the presence of p53 enables the expression modulation of at least 1000 genes in cells infected with IAV [43]. Thus, there is a possibility that the activation of p53 and p53-dependent apoptosis is not a cell-defense mechanism but a tactic used by a virus to spread more efficiently. In the light of the role of apoptosis in the propagation of IAV, it would appear that the virus hijacks p53 to spread itself more efficiently. Surprisingly, however, the authors [41] who observed that the infection of cells with IAV leads to apoptosis mediated by the activation of p53 also perceived that the presence of p53 leads to the reduction of IVA titers. Some of these scientific observations suggested that interferon signaling was impaired in p53-deficient cells. Hence, it was hypothesized that p53 promotes interferon signaling, which in turn interferes with viral replication. Considering the papers cited hereinabove, there are disturbing discrepancies concerning the role of p53 in the replication of IAV in cells cultured in vitro. Conducting in vivo experiments helps to settle the matter.

The role of p53 in the promotion of interferon signaling was directly studied by Yan et al. [44] and Zhu et al. [45]. Mice with wild-type p53 (p53WT) or p53 knockout mice (p53KO) were infected with IAV and lung samples were examined for virus titer and gene expression profiles. When compared to the p53WT mice, the p53KO mice were more susceptible to IAV infection. Moreover, the p53KO mice had significantly changed expression of a range of genes associated with interferon signaling, e.g., the gene for interferon gamma, the gene for interferon regulatory factor 7 (IRF7), and genes for some cytokines and chemokines [44]. In another study, the upregulation of tested interferon-stimulated genes in p53-deficient cells was attenuated following exposure to IAV and interferon. Thus, p53 plays an essential role in the enhancement of interferon signaling against IAV infection [45]. Furthermore, there are data indicating that not only does p53 promote innate immunity but it also stimulates the adaptive immune response. P53 promotes expression of major histocompatibility complex class I (MHCI) on the cell surface. MHCI is responsible for the presentation of antigens for cytotoxic T lymphocytes (e.g., fragments of viral proteins, cancer antigens) on the cell surface. Thus, diminished expression of MHCI molecules in p53-deficient cells may attenuate their recognition by the immune system once infected by a virus or when oncogenically transformed [46].

P53 was also found to interact with various proteins coded by human immunodeficiency virus type 1 (HIV-1) and herpes simplex virus 1 (HSV-1). The influence of p53 on the HIV-1 life cycle is complicated, since various viral proteins may either stimulate or inhibit p53 activity. Overexpression of p53 reduces the transcription from the major gene-regulatory element of HIV-1, the LTR sequence. In the case of HSV-1, it unexpectedly seems that p53 promotes its replication, which has been demonstrated using the p53 knockout mouse model. However, judging by the influence of p53 on individual viral proteins, the picture is not so clear-cut, because p53 promotes the expression of viral protein (ICP27) required for replication at an early stage. On the other hand, however, p53 promotes degradation of the ICP0 protein, which is also essential for viral replication, and ICP0 additionally represses the host immune response (reviewed by Aloni-Grinstein et al. [30]). In experiments with p53 knockout mice demonstrating the positive role of p53 in the replication of HSV-1, the animals were inoculated intracranially with the virus [47]. Thus, the influence of p53 on HSV-1 replication in more physiological conditions remains to be determined.

In recent years, the world suffered the COVID-19 pandemic caused by the spread of various strains of the SARS-CoV-2 coronavirus. However, the harbinger had already appeared in 2002 with the emergence of the SARS-CoV coronavirus, which caused SARS cases, mostly in Asia. Since that time, coronaviruses have been in the spotlight and it is not surprising that researchers, even before COVID-19, started studying the relationship between coronavirus and the p53 pathway. The papain-like proteases (PLPs) of the coronavirus were soon discovered to be suppressors of the innate immune response. One of these enzymes, PLP2 of the human coronavirus NL63, stabilizes the antagonist of p53—the MDM2 protein. Thus, NL63 has a potent mechanism to antagonize the p53 pathway. Interestingly, in the same study, p53 was reported to directly stimulate the IRF7 gene, which codes interferon regulatory factor 7 [48]. When activated by phosphorylation, this transcription factor promotes the expression of the potent antiviral protein interferon beta (IFNβ). Thus, the NL63 coronavirus uses PLP2 to antagonize the antiviral innate immune pathway p53-IRF7-IFNβ. Similar conclusions were drawn from experiments with SARS-CoV. The papain-like protein coded by the virus in question stabilizes the cellular protein RCHY1, which promotes p53 degradation. Other coronaviruses (MERS-CoV, HCoV-NL63) also code for proteins that utilize cellular RCHY1 to degrade p53 [49]. Does p53 have the ability to antagonize coronavirus replication? In principle it does, because the coronavirus makes an effort to inactivate it. This has been experimentally demonstrated in vitro, using a model system of p53-proficient and p53-deficient HCT116 cells engineered to express the receptor for SARS-CoV. The replication of the virus was much more efficient In the p53 knockout cells [49]. To appreciate the role of p53 in preventing the replication of coronaviruses, the authors referred to this protein as “an antiviral factor p53”. Unsurprisingly, SARS-CoV-2 also encodes a protein that antagonizes p53. In an experiment using a signaling pathway reporter screen, Kumar et al. [50] found that the main viral protease nsp5 may repress transcriptional p53 activity. On the other hand, p53 has been found to inhibit the production of SARS-CoV-2 virus particles in culture cells [50].

**Figure 2:**
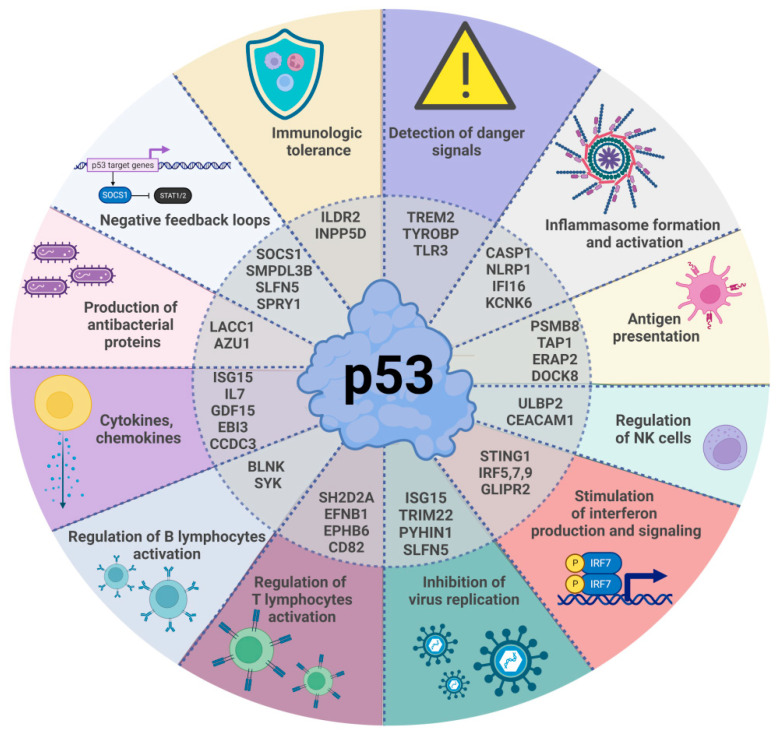
Immune-related processes regulated by p53 through activation of the indicated genes. The list of genes is far from complete, partly because p53 regulates expression of many genes with unknown function, which potentially may participate in immunity. Regulation of some genes by p53 is well-documented, whereas the involvement of p53 in regulation of other genes is only currently being discovered—see text. Created with BioRender.com (accessed on 14 February 2023).

P53 may antagonize the replication of several types of viruses including those that can cause pandemics (influenza A virus, coronaviruses). What are the mechanisms utilized by this “antiviral factor p53” to inhibit the spread of viruses? Some of them have already been hinted at: inhibiting the transcription of the SV40 large T-antigen, promoting the production of interferons, promoting the expression of the MHCI complex on the cell surface. In the following section we provide more details of the mechanisms utilized by p53 to boost antiviral or more general antimicrobial immunity. We review these mechanisms based on individual reports and high-throughput studies, showing that p53 may upregulate the expression of immunity genes. This illustrates the versatility of p53 in engaging various cellular mechanisms of antimicrobial defenses. We were prompted to conduct this review by our recent transcriptomic analysis of the A549 lung cancer cell line exposed to two substances, actinomycin D and nutlin-3a (A+N), which synergize in the activation of p53. We noticed that many genes activated by A+N, both the known and the candidate targets of p53, code for immunity-related proteins [7]. Learning the functions thereof, we were surprised by the diverse roles they play in immunity. Hence, we decided to arrange them in several groups to provide a better description of the actual or potential functions of p53 in immunity (Figure 2). We also referred to a database summarizing high-throughput transcriptomic searches for the p53 target genes [8]. Based on the number of studies reporting a gene as a p53 target, it can be estimated whether the gene is a common p53 target activated by various stress factors in most cell lines or whether the regulation thereof is restricted to a particular cell type or stress condition. However, it is advisable to consider that many transcriptomic studies were performed on the same cell lines (e.g., U-2 OS, HCT116, and MCF7 cells frequently appear in the database), the cells were exposed to the same p53 activator (e.g., nutlin-3a), or the studies were performed using low-sensitivity microarray techniques. Hence, we believe that a small number of positive reports does not automatically mean that the gene is not regulated by p53 in a subset of cell lines or under specific stress conditions.

## 5. Inflammasome Formation and Activation

Inflammasomes constitute cytosolic, multimeric structures consisting of at least two protein types—pattern recognition receptors (PRR) and caspases (inactive procaspases, e.g., procaspase-1 in the best-studied inflammasomes). PRRs recognize various threat signals such as pathogen-associated molecular patterns (PAMPs) or damage-associated molecular patterns (DAMPs). PAMPs are molecules created by pathogens, e.g., bacterial toxins, peptidoglycans or lipopolysaccharides of bacterial cell walls, and viral DNA or RNA. Examples of DAMPs include K^+^ efflux, production of reactive oxygen species, and rupture of lysosomes [51].

Detection of DAMPs or PAMPs triggers conformational changes within the inflammasome proteins, leading to proteolytic cleavage of procaspase-1, which folds into its active form and cleaves its substrates. One of these, gasdermin D, makes pores within the cell membrane, which leads to cell death known as pyroptosis. This form of regulated death triggers an inflammatory response, as the leaked cell content is a threat signal (e.g., extracellular ATP) to neighboring cells. Furthermore, active caspase-1 cleaves and activates precursors of the inflammatory cytokines IL-1β and IL-18. Generally, most of what is known about inflammasomes derives from studies utilizing macrophages, yet the components of inflammasomes have been recently detected in epithelial barrier tissues, and these models h poorly studied [52].

Treatment of cells with actinomycin D and nutlin-3a triggers a substantial induction (more than 1000-fold) of the procaspase-1 gene *CASP1*, and these two compounds strongly synergize in this process [53]. Earlier studies demonstrated that *CASP1* is regulated by p53 [54]. Interestingly, CASP1 appears to play an important role in dengue-virus-induced p53-mediated apoptosis [55]. This suggests that infection with dengue virus activates p53, which in turn stimulates the *CASP1* gene. The elegant study conducted by Schlereth et al. [56] revealed that the p53 binding to the response element in the *CASP1* gene strongly depends on the cooperativity of p53 monomers. The mutational inactivation of cooperativity fails to compromise the expression of p53 targets inhibiting the cell cycle (e.g., *CDKN1A*) but does inhibit the expression of *CASP1* and other cell-death-inducing genes. Thus, it may be hypothesized that *CASP1* is activated by p53 in a manner which promotes cooperative binding of p53 monomers. Such modifications may be induced only by particular stress factors, e.g., treatment with A+N. Consistent with this, *CASP1* has been found to be upregulated by p53 in only 16 out of 57 transcriptomic studies (Table 1). 

The p53 protein stimulates the expression of other components of inflammasomes—IFI16 [62]. The expression thereof may also be stimulated by interferon alpha [53]. This gene has been found to be stimulated by A+N (17-fold) (Table 1). Transcriptomic studies have provided apparently contradictory results, as in eight studies *IFI16* has been found upregulated by p53, whereas in ten studies p53 has downregulated that gene [8]. In six6 studies showing the downregulation of *IFI16*, the stress factor was cell senescence. Thus, in case of this gene, the direction of regulation by p53 is apparently stress- and/or cell-type specific. Perhaps the *IFI16* gene is suppressed in cells that undergo p53-dependent senescence. In a different set of our data, actinomycin D and nutlin-3a were strongly synergized in the activation of this gene, which was clearly attenuated in p53-deficient cells. Thus, p53 positively regulates expression of *IFI16* in A549 cells exposed to A+N [53]. *IFI16* acts as a sensor of foreign DNA. In contrast to other inflammasomes that are present in cytosol, inflammasomes with *IFI16* may sense pathogen-derived molecules (e.g., Kaposi sarcoma-associated herpesvirus) in the cell nucleus [72]. A component of the first identified inflammasome, NLRP1 (NALP1) recognizes bacteria-derived molecules including the lethal toxin *Bacillus anthracis* [51]. Recent studies have indicated that not only does NLRP1 detect bacterial proteins, but it also identifies double-stranded RNA of the replicating Semliki Forest virus [52]. *NLRP1* is strongly activated by A+N (500-fold), with strong synergy between actinomycin D and nutlin-3a. In p53-deficient cells, its activation by p53 was distinctly attenuated and its promoter was activated by the wild type but not by the p53 mutant [53]. It was identified as a p53 target in 36 out of 57 high-throughput studies (Table 1), which further supports the notion that p53 activates this gene.

Interestingly, our transcriptomic search revealed another inflammasome-related gene—*KCNK6*—that may be activated by A+N (Table 1). No individual study has reported the association thereof with p53, however, its upregulation by the said protein was detected in 27 out of 57 high-throughput studies (Table 1), which makes it a strong candidate for a p53-regulated gene. By promoting the expression of *KCNK6*, p53 may regulate the activation of inflammasomes by means of another mechanism. *KCNK6* is a potassium channel, which mediates the K^+^ efflux triggering activation of inflammasomes [73]. In conclusion, p53, at least when exposed to particular stress conditions, activates the genes coding for inflammasome caspase (CASP1), pattern recognition receptors (NLRP1, IFI16), and the protein that mediates potassium efflux which is critical for inflammasome activation (KCNK3). Despite the expression of all known components of at least two types of inflammasomes (CASP1, IFI16, PYCARD, CASP1, and NLRP1), we did not observe activation of caspase-1 in the cells exposed to A+N [53]. We hypothesized that the cells are primed for pyroptosis, but, due to the sterile culture conditions, the crucial trigger was missing (e.g., foreign DNA, long double-stranded RNA, bacterial proteins). However, our treatment modality (A+N) induces the expression of inflammasome components in cancer cells and provides a model for studying inflammasome activation in cells other than macrophages.

## 6. Stimulation of Interferon Production and Signaling

**Figure 3:**
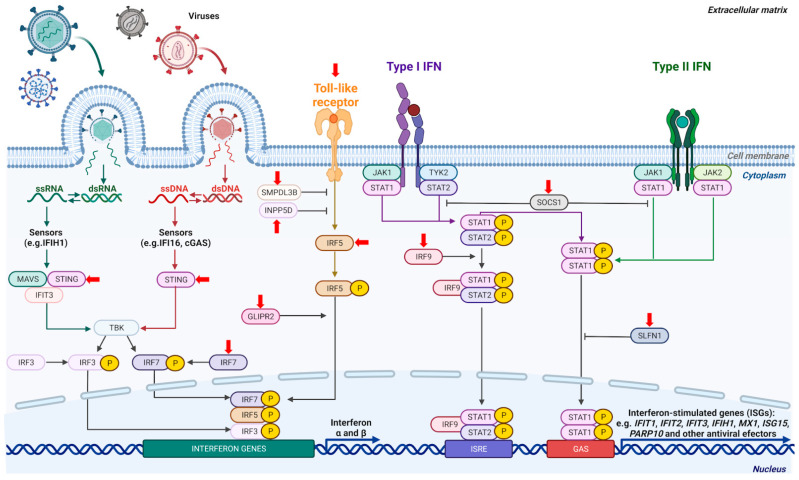
The involvement of p53 in activation of interferon genes, of interferon-stimulated genes, and in feedback loops regulating these processes. Red arrows mark the genes positively regulated by p53. ISRE—interferon-stimulated response element, a DNA sequence that confers responsiveness to type I interferons. GAS—interferon-gamma-activated site, a DNA sequence element through which interferon gamma can activate transcription. For clarity, the signaling from toll-like receptors is oversimplified, e.g., some of them are localized in the cell membrane, whereas others are localized in the endosome membrane. The exact localization of GLIPR2 in this signaling pathway is not known. STING protein can also recognize foreign DNA in the cell nucleus. The entry method of viruses into cells is also only schematically represented. Adapted from “Interferon Pathway”, by BioRender.com (2023). Retrieved from https://app.biorender.com/biorender-templates accessed on 14 February 2023.

Interferons were discovered and identified as strong antiviral molecules in the late 1950s. Since that time, their activation as well as their influence on cells has been extensively studied (reviewed by Schneider et al. [74]). To better comprehend their functioning, it seems convenient to divide this elaborate signaling system into two parts—the induction of interferon-encoding genes and the response of cells to interferon accumulation. A detailed description of this signaling system is beyond the scope of this review. We present here some basic information that will help to develop an understanding of the interplay between interferons and p53 signaling pathways (Figure 3).

The induction of interferon genes (this mostly concerns type I interferons—see below) commences with the detection of an ongoing viral infection. The presence of a virus is detected by sensors (pattern recognition receptors), e.g., IFIH1 detects viruses with RNA genomes, whereas IFI16 detects viruses with DNA genomes. This function of IFI16 does not rely on its association with caspase-1. There are also other sensors of viral genomes (e.g., toll-like receptors). The signals from sensors, through a series of molecular interactions and protein modifications by phosphorylation, lead to the activation of interferon genes and secretion of interferons. For instance, the TBK kinase phosphorylates IRF3 and the IRF7 transcription factors, which directly bind to gene regulatory elements of the interferon genes. The STING1 protein is involved in signaling from both DNA and RNA sensors. The IFIT3 protein antagonizes the translation of mRNAs of some viruses, but it also helps in the activation of TBK. MAVS constitutes a mitochondrial antiviral-signaling protein, which cooperates with both IFIT3 and STING1 in the activation of TBK [74,75,76].

Once interferons are secreted into the extracellular space, they bind to their cognate receptors. It is a complicated system, as there are more than 20 interferons divided into 3 types. The type I family consists of 17 distinct proteins, the best studied examples of which are IFNα1 and IFNβ1. Type II consists of a single member, IFNγ. Type III consists of four members, IFNλ1- IFNλ4. Type I and type III expressions are induced by pattern recognition receptors, while INFγ expression is stimulated by mitogens and cytokines. Type I IFNs may be produced by most cell types. The production of IFNγ is primarily restricted to T cells or NK cells, however, every cell may respond to it. Interferons λ are mainly produced by monocytes, macrophages, and dendritic cells. Binding of IFN to its receptor activates a pair of JAK and TYK kinases, which phosphorylate transcription activators of the STAT family (STAT1-STAT6), which can homo- and heterodimerize and activate various combinations of interferon-stimulated genes (ISGs). Type I interferons, e.g., IFNα1, induce phosphorylation of the STAT1 and STAT2 transcription factors, which heterotrimerize with the IRF9 protein and activate the expression of a subset of interferon-stimulated genes (e.g., strong activation of the *MX1* gene). IFNγ binds to its own receptor and induces phosphorylation of STAT1 molecules, which form homodimers and activate a different set of genes (e.g., strong activation of the *IRF1* gene). Thus, the sets of genes stimulated by type I and type II interferons overlap but are not identical.

There are both positive and negative feedback loops built in this system. For instance, the *IFIH1* gene, which is stimulated by interferon, codes a protein that acts as a viral RNA sensor, triggering the expression of interferon genes and resulting in a positive feedback loop. On the other hand, IFNγ induces the expression of the SOCS1 protein, which acts in a negative feedback loop, reducing the level of phosphorylated STAT1 (reviewed by Schneider et al. [74]).

The connection between p53 and interferon signaling pathways has already been observed and reviewed [6]. The p53 gene was transcriptionally induced by IFNβ, but induction at both the mRNA and protein levels was relatively small (three-fold) [77]. Interestingly, we did not observe the upregulation of p53 in A549 cells exposed to IFN-α1 [53]. Thus, the transcriptional upregulation of the p53 gene in response to type I interferons may depend on cell and/or interferon type. P53 may be upregulated by interferon but, on the other hand, p53 may promote the expression of interferon-stimulated genes because p53 activates the expression of the gene coding for the IRF9 transcription factor [65], which forms a heterotrimer with phosphorylated STAT1 and STAT2 (Figure 3). Unexpectedly, only 4 out of 57 high throughput studies found *IRF9* to be activated by p53, while in 6 studies the gene in question was downregulated by p53 [8]. We also observed upregulation of *IRF9* expression in A549 cells exposed to A+N (Table 1). Moreover, in our unpublished RNA-Seq data we have found the activation thereof following the A+N exposure in the A549 (an independent experiment) and the NCI-H460 cell lines derived from lung cancer and in the A375 cells derived from melanoma. Interestingly, we observed only a slight activation of IRF9 in U-2 OS cells exposed to A+N. It appears that IRF9 constitutes an example of a gene whose activation by p53 is cell-type- and stress-dependent. In our opinion, due to the crucial role of IRF9 in the activation of interferon-stimulated genes, its plausible regulation by p53 deserves more attention.

P53 activates the expression of another interferon regulatory factor—the IRF7 protein, which if phosphorylated by TBK (Figure 3) directly activates the expression of type I interferons [48]. It is activated in cells exposed to A+N and was found to be a p53 target in only 6 out of 57 high-throughput studies (Table 1). P53 also activates *IRF5* [64] coding a transcription factor, activating genes for type I interferons. However, IRF5 operates downstream a different class of pattern recognition receptors (Figure 3) known as Toll-like receptors [78]. This gene is activated by A+N and is more frequently found in a transcriptomic search for p53 targets (Table 1). Thus, at least in some cell types, p53 activates three interferon regulatory factors: IRF9, IRF7, and IRF5, whereby it may both promote the production of interferons and enhance the expression of interferon-stimulated genes. Another protein, called GAPR-1 (gene name *GLIPR2*), participates in the stimulation of type I interferons initiated by toll-like receptors. This protein has been poorly studied and there is only one report linking it with the activation of interferon genes [79]. *GLIPR2* is activated by A+N and the analysis by Fischer et al. [8] supports this hypothesis, as it is frequently found as a p53-activated gene in high-throughput searches (Table 1).

A very interesting protein involved in interferon-related innate immunity is called STING1. The name denotes a stimulator of interferon genes, which describes its function. However, it appears not only to play an important role in innate immunity and antiviral defense, but may also constitute a target for anticancer therapy [80]. Hence, the number of studies on STING1 has been rapidly growing in recent years. We observed that actinomycin D and nutlin-3a strongly synergize in the activation of STING1, which is visible at both the mRNA and protein levels. In p53-deficient cells, the activation of the STING1 mRNA and protein are significantly attenuated. Furthermore, what may be relevant for cancer therapy is the fact that STING1 may also be strongly activated by camptothecin, which is a precursor of anticancer drugs [53]. Thus, our observations indicate that STING1 is regulated by p53 (at least indirectly). The review conducted by Fischer et al. [8] showed that *STING1* was identified as a p53 target in almost half of the studies (Table 1), which makes it very likely to be a p53 target across cell types and treatment conditions. In conclusion, p53 by stimulating the expression of STING1, IFI16, IRF5, IRF7, and GLIPR2 may boost the production of type I interferons, and via the activation of IRF9 may increase the expression of interferon-stimulated genes. Considering the comprehensive positive impact of p53 on interferon signaling, it is not surprising that viruses inactivate this protein.

## 7. Detection of Danger Signals

The innate and adaptive immune response begins with the detection of a pathogen. We have already mentioned the proteins that form inflammasomes, which identify bacteria or viruses (e.g., IFI16, NLRP1). In this section, we discuss proteins located elsewhere in immune-related signaling systems. We found that treatment of A549 cells with A+N stimulates the expression of the signaling pathway elements, starting from the TREM2 receptor on the plasma membrane and encompassing TYROBP (a binding partner of TREM2), the SYK kinase, and the BLNK adapter protein (Table 1). In our experiment, the accumulation of all these proteins was clearly attenuated in p53-deficient cells [17], suggesting that p53, at least indirectly, stimulates their expression. In the case of TREM2, p53 directly activates this gene [17]. *BLNK* and *SYK* have been identified by other researchers as p53-regulated genes [57,81]. The physiological significance of upregulation of these proteins by p53 remains elusive. TREM2 is expressed mainly in macrophages and in microglia, and its polymorphisms are strongly linked to the risk of Alzheimer’s disease [82]. We detected that not only may TREM2 be upregulated by A+N, but the upregulation thereof (in some cell lines) may also constitute an effect of nutlin-3a acting alone [17]. TREM2 binds various ligands including bacterial products, DNA, LDL (low density lipoproteins), Apo E, and pathological β-amyloid oligomers. TREM2 acts as a negative regulator of signaling in macrophages and dendritic cells, beginning with toll-like receptors. Consequently, TREM2-deficient bone-marrow-derived dendritic cells produce increased type I interferon and inflammatory cytokines in response to TLR agonists (reviewed by Qiu et al. [82]). Some observations suggest that the imbalance between TREM2 and TLR4 (toll-like receptor 4) signaling in favor of TLR4 may contribute to the development of Alzheimer’s disease [83]. Whether the objective of p53-induced TREM2 is to prevent excessive pro-inflammatory signaling remains to be determined.

TLR4 is a cell-surface receptor that recognizes bacterial polysaccharides, whereas TLR3 senses viral and endogenous double stranded RNA in endosomes. Once activated, TLRs initiate signaling cascades that culminate with the secretion of pro-inflammatory cytokines, including TNF-α and type I interferons. TLR3 and TLR4 signaling, despite some similarities, may lead to different outcomes [84]. The TLR3 gene is positively regulated by p53 [68]. The gene is activated by A+N and has frequently been found as a p53 target in transcriptomic studies (Table 1). Thus, stimulating the expression of this receptor is another mechanism by means of which p53 promotes the expression of type I interferons in cells infected by RNA viruses (Figure 3).

## 8. Antigen Presentation

The adaptive immune system constantly monitors the protein content of cells. The peptides derived from internal proteins are presented to lymphocytes by surface proteins called major histocompatibility complex class I (MHC class I). The cytosolic or nuclear proteins are degraded into peptides in ordered multiprotein structures called proteasomes. Subsequently, these peptides are transported into the lumen of the endoplasmic reticulum (ER) by transport proteins called TAP. In the ER, the peptide assembles with the newly synthesized MHC class I heavy chain and β_2_ microglobulin. This assembly involves transient interactions with more proteins. Finally, the mature complex of MHC class I and the peptide is released from the ER and transported to the cell surface via the constitutive secretory pathway. Should the peptide be judged as foreign by the immune system, the cells bearing the peptide in the complex with MHC class I are killed by cytotoxic T lymphocytes. In such a way, the adaptive immune system kills virus-infected cells and cells producing cancer-specific antigens [85]. We observed that p53 may activate several genes that code proteins involved in this process. One of the examples is *PSMB8* upregulated by A+N (Table 1). The PSMB8 protein constitutes an element of special-purpose proteasomes called immunoproteasomes, which are induced by interferon γ [86]. The function thereof is the processing of MHC class I peptides. We found no individual study reporting the activation of the PSMB8 gene by p53, and this gene was considered to be activated by p53 in 10 reports from high-throughput studies. However, in five reports it was found to be downregulated by p53 [8]. Thus, the regulation of this gene by p53 is not proven, yet it seems plausible in at least some cells or under certain stress conditions and definitely deserves further research.

Another gene that we found to be upregulated by A+N is *TAP1* (Table 1). The TAP1 protein in complex with TAP2 mediates unidirectional translocation of peptide antigens from cytosol to the ER for loading onto MHC class I molecules [87]. This gene was identified as a p53 target [67], and most of the transcriptomic studies (50/57) report it as regulated by p53 (Table 1). Thus, the positive regulation of this gene by p53 is well documented.

The *ERAP2* gene involved in antigen presentation is also upregulated by A+N, and is frequently considered as a p53 target in transcriptomic studies (Table 1). It codes for aminopeptidase located in the ER, which is involved in trimming peptides present on MHC class I. This trimming is essential to fit longer peptides into the groove formed by MHC [88]. Therefore, by stimulating the expression of ERAP2, p53 promotes formation of the mature peptide–MHCI complex. Interestingly, p53 activates a gene for the related protein ERAP1, which promotes MHCI expression on the cell surface [46].

A very interesting gene associated with antigen presentation is *DOCK8*, which codes for a guanine nucleotide exchange factor involved in signal transduction. The gene is expressed in hematopoietic cells, but also in non-immune tissues such as lung, pancreas, and kidney. Interestingly, *DOCK8* deficiency in humans due to bi-allelic mutations results in combined immunodeficiency characterized by recurrent viral infections and early-onset malignancy. Patients also suffer from severe food allergies. In the absence of the DOCK8 protein, dendritic cells, which constitute the major antigen-presenting cells, fail to accumulate in the lymph node parenchyma for T-cell priming. This naturally occurring gene knockout in humans (more than 200 cases have been reported worldwide) indicates that *DOCK8* is critical in fighting viral infections and preventing cancer formation, especially in the skin (reviewed by Kunimura et al. [89]). In our experiments, *DOCK8* was upregulated 14-fold by actinomycin D and nutlin-3a, and a review of transcriptomic data also demonstrated frequent upregulation of this gene by p53 (Table 1). These very convincing data indicate that p53 promotes antiviral defense by positively regulating the expression of *DOCK8*, which apparently helps antigen-presenting cells migrate to lymph nodes in order to present antigens to T cells.

## 9. Regulation of Natural Killer (NK) Cells

NK cells are effector cells of the innate immune system. They sense their environment by means of cytokine receptors, which promote their proliferation and help to guide them to the site of inflammation. Then, another type of receptor instructs them on whether encountered cells are healthy, infected, or on the way to tumorigenic transformation. The ligands for these receptors that are present on target cells either activate or inhibit the effector activity of NK cells. Based on the number of activating and inhibiting ligands on target cells, NK cells decide whether a cell must be executed or saved. One of the strongest signals for killing is a loss of MHC class I molecules from target cells. MHC molecules present foreign antigens to cytotoxic T cells. Therefore, in order to hide their presence, viruses remove MHC molecules from the cell surface. The immune system is prepared for this and sends NK lymphocytes to remove MHC-deficient cells. However, the loss of MHC molecules is not the only signal that activates NK cells. There are more than 10 other activating or inhibiting receptors present on NK cells, which monitor the health of target cells by sensing the number of ligands on the surface thereof [90]. One of the activating ligands is the ULBP2 molecule, which binds to the activating receptor NKG2D. P53 is a direct activator of the *ULBP2* gene, as demonstrated by two independent reports [70,71]. The activation of p53, e.g., by DNA damage, promotes the expression of ULBP2 on stressed cells, which makes them better targets for the effector function of NK cells. The expression of ULBP2 is also stimulated (14-fold) by A+N, and 23 transcriptomic studies have identified this gene as a p53 target (Table 1). Thus, the evidence that p53 stimulates the expression of ULBP2 is strong. Yet, the role of p53 as a positive regulator of activating signals for NK cells seems to be more complicated than one may expect, because p53 also positively regulates the expression of the *CEACAM1* gene [60]. The gene in question encodes a member of the carcinoembryonic antigen family—a cell adhesion molecule. Its expression on cancer cells causes intracellular retention of various proteins that act as activating ligands for NK cells [91]. Another study demonstrated that should this protein be expressed on both cancer cells and NK cells, it mediates the inhibition of cancer cells’ destruction by NK cells [92]. Thus, the expression of CEACAM1 promotes the escape of cancers from immune surveillance. Since CEACAM1 has a robust inhibitory effect on NK-mediated cancer cell killing, cancer immunotherapy strategies targeting this protein have been considered [93]. Why does the p53 tumor suppressor promote the expression of such a strong antagonist of the immune response? Does *CEACAM1* really constitute a p53 target? We detected a very strong (150-fold) activation thereof by A+N, and it is also frequently found in transcriptomic data as a gene positively regulated by p53 (Table 1). Thus, this gene is considered very likely to be a target of p53, which leaves us puzzled as to why the tumor suppressor promotes the expression of an antagonist of the immune response against cancer cells.

## 10. Direct Inhibition of Virus Replication

Interferons stimulate the expression of many genes that code for proteins directly interfering with virus replication. Interestingly, some genes stimulated by type I or type II interferons are also activated by p53. For instance, p53 and IFNγ both stimulate the expression of *PML* [94,95] and *DR5* [96,97]. Hence, p53 and interferons may inhibit the replication of viruses via similar mechanisms. One of the first interferon-stimulated genes to be discovered is named *ISG15* (interferon-stimulated gene 15) [31]. It is an abundant protein with many antiviral functions. Its expression is stimulated by both type I and type II interferons. The gene codes for a small protein that, like ubiquitin, may be attached to other proteins in the process referred to as ISGylation. Furthermore, as an example of multiple functions in one protein, free ISG15 may be secreted and act as a cytokine. ISGylation of viral proteins impairs their functions, hence many viruses counteract this modification. Unexpectedly, as far as ISG15 is concerned, many of the experiments conducted in mouse models contradict the conclusions drawn from studies of human cells. This constitutes another example in which the results of experiments with mice cannot be extrapolated to humans [21]. As early as 2001, ISG15 was found to be regulated by p53 [66]. ISG15 is strongly upregulated by A+N and it has also been frequently considered a p53 target in other transcriptomic studies (Table 1). Thus, the positive regulation of this versatile antiviral gene by p53 is well documented.

Another multitasking antiviral gene regulated by interferons and p53 is TRIM22. This gene is upregulated by both type I and type II interferons. TRIM22 has been found to interfere with Sp1-dependent transcription of HIV-1 genes, and prevented the binding of Sp1 to the viral promoter in the LTR sequence. A different mechanism is used by TRIM22 to counteract the replication of influenza A virus. The protein has E3 ubiquitin ligase activity, which catalyzes the ligation of pre-activated ubiquitin to the lysine residues of the essential nucleoprotein (NP) of the virus. In addition, TRIM22 counteracts the replication of other viruses with either RNA or DNA genomes (e.g., hepatitis C virus, respiratory syncytial virus, herpes simplex virus type-1) (reviewed by Pagani et al. [98]). TRIM22, alternatively named Staf50, has been found to be directly regulated by p53 [69]. Exposure to A+N stimulated its expression 60-fold, which is consistent with its being identified as a p53 target in 47 high-throughput studies (Table 1). Thus, p53 counteracts the replication of a wide range of viruses by stimulating the expression of the TRIM22 ubiquitin ligase.

The *PYHIN1* gene (alternatively named IFIX, interferon-inducible protein X) belongs to the same protein family as the aforementioned IFI16; both proteins may sense foreign DNA. The PYHIN1 protein accumulates in response to interferon α and negatively regulates MDM2, which is an antagonist of p53. Thus, in principle, prolonged treatment with interferon α may lead to the activation of p53 [99]. Our study, employing a different model, found no accumulation of p53 in cells exposed to this cytokine [53]. If this mechanism exists, it may be cell-type specific. Interestingly, we demonstrated that the expression of *PYHIN1* is strongly activated by A+N (365-fold, Table 1); this strong fold-change is associated with a very low expression of the gene in unstressed cells [7]. Transcriptomic data does not suggest that this gene is universally activated by p53 (Table 1), yet it remains possible that PYHIN1 may be utilized by p53 for antiviral defense, at least under some conditions or in certain cell types. IFIX suppresses viral gene expression during HSV-1 infection. The virus mounts a counterattack by promoting the degradation of PYHIN1 [100]. Overall, PYHIN1 is a poorly-studied protein, but its plausible involvement in p53-mediated antiviral defense merits attention.

Another protein coded by a type I interferon-stimulated gene is SLFN5 (Schlafen Family Member 5) [101]. SLFN5 is another poorly-studied protein, which has been found to regulate negatively the replication of at least two viruses. SLFN5 binds to the HSV-1 DNA to repress transcription by limiting the accessibility of RNA polymerase II to viral promoters. To counterattack, the virus protein ICP0, which is an E3 ubiquitin ligase, targets SLFN5 for degradation [102]. Other authors have reported that the overexpression of SLFN5 inhibits the replication of another virus—HIV-1. Specifically, SLFN5 markedly decreases the transcriptional activity of HIV-1 long terminal repeat via binding to sequences in the U5-R region [103]. Hence, SLFN5 antagonizes the life cycle of at least two different viruses by directly repressing the transcription of their genes. Actinomycin D and nutlin-3a upregulate this gene 10-fold and *SLFN5* is frequently found as a p53 target in transcriptomic data (Table 1). Thus, p53 promotes antiviral defense in part by upregulating the SLFN5 gene, which, interestingly, may also play a role as a negative feedback loop element in the interferon signaling pathway (see below).

## 11. Regulation of the Activity of T Lymphocytes

T-cell activation is a major event in adaptive immunity. It enables a T lymphocyte to acquire the ability to kill infected cells or to stimulate other cells of the immune system. The key activation step involves a specific interaction between the T-cell receptor and the antigen on MHC class I or class II molecules on target cells. Yet, this interaction is not sufficient, for both cells must interact by the so-called co-stimulatory proteins. One of these co-stimulatory proteins is CD82, which is expressed on target cells as well as on T lymphocytes [104]. Its activation by p53 mediates the suppression of metastasis [59]. In our experiments, it was one of the genes most strongly activated by A+N (more than 1000-fold), and transcriptomic studies have demonstrated its regulation by p53 in 45 reports (Table 1). Both functions of CD82 (inhibition of metastasis and activation of T cells) may be associated with its ability to modify the cytoskeleton [105].

Exposure to A+N strongly upregulated the expression of *SH2D2A*—a gene (Table 1) that encodes the T cell-specific adapter protein involved in the regulation of T-cell activation. Its expression has been also found in NK cells, endothelial cells, and epithelial cells (reviewed by Borowicz et al. [106]). The adapter proteins lack catalytic activity, but they guide various enzymes to modify their targets through protein–protein interactions. The function of the gene in question has been poorly studied. Several interacting partners have been identified. Mice lacking its expression are apparently normal, but they spontaneously develop systemic autoimmune disease as they grow older, although this has not been confirmed using a different model. Interestingly, mice deficient in this gene displayed reduced clearance of murine cytomegalovirus in the spleen. SH2D2A also plays a unique role in VEGFR (vascular endothelial growth factor receptor) signaling, as it controls the opening of endothelial adherens junctions and, consequently, vascular permeabilization. Polymorphism in the SH2D2A promoter region has been associated with increased susceptibility to autoimmune diseases including multiple sclerosis, juvenile rheumatoid arthritis, chronic inflammatory demyelinating polyradiculoneuropathy, and Sjogren’s syndrome (reviewed by Borowicz et al. [106]). *SH2D2A* has been identified as a p53 target in 20 transcriptomic reports (Table 1). Given this result and the strong upregulation of SH2D2A by A+N, this gene appears to be a likely target of the p53 tumor suppressor. By stimulating the expression of this adapter, it may modulate post-translational modifications of various proteins downstream of T-cell receptor or VEGF receptor signaling, which in turn may change the biological signaling outcome.

Eph kinases are the largest family of receptor tyrosine kinases. Their ligands are called ephrins, and are also cell surface molecules. Thus, the binding of an ephrin ligand to its receptor requires close contact between the cells. Among other things, this signaling system is important for the functioning of immunity. The gene (*EFNB1*) coding for ephrin-B1 is strongly activated in cells exposed to A+N (Table 1). The EFNB1 receptor enhances signaling from T-cell receptors (TCR). It appears that EFNB1 reduces the threshold of T-cell response to antigen stimulation [107]. However, an interesting observation regarding this signaling should be noted. At low concentrations, EFNB1 co-stimulated the signaling from TCR, whereas at high concentrations it strongly inhibited it [108]. Thus, should p53 activate EFNB1 expression in the microenvironment of stressed cells, it seems difficult to predict the influence thereof on cell activation, but it is plausible that the effect is inhibitory. In transcriptomic studies, the *EFNB1* gene was activated by p53 in 46 reports (Table 1). Therefore, this gene constitutes a very likely target of p53. However, the mechanism of plausible regulation of TCR signaling by p53 via ephrin B1 requires further investigation.

Ephrin type B receptor 6 coded by the EPHB6 gene was also upregulated by A+N (Table 1). This protein lacks the tyrosine kinase activity present in other ephrin receptors. *EPHB6* is primarily expressed in thymocytes and a subpopulation of T cells, which suggests that it may be involved in the regulation of T-lymphocyte functions. Overexpression of EPHB6 in T cells and stimulation with its ligand, ephrin-B1, results in inhibition of TCR-mediated JNK activation but not the MAPK pathway [109]. Thus, this receptor, even if not catalytically active, may modulate the T-cell response by promoting one signaling pathway over another, starting with the TCR. When expressed in cells other than T lymphocytes, it plays a similar role in relation to other tyrosine kinase receptors, and results in, for example, reduced motility and invasion of cancer cells (reviewed by Strozen et al. [110]). Transcriptomic studies do not provide a clear answer to whether the gene in question constitutes a p53 target, as nine studies have found it to be activated by p53, but six studies have found it to be downregulated by p53 [8]. Our unpublished results suggest that the direction (up or down) of gene regulation by p53 may depend on the level of gene expression under stress-free conditions. Should a p53-regulated gene be expressed at a very low level under control conditions, p53 activation may stimulate its expression; should the same gene be expressed in another cell type at a high level under control conditions, p53 activation may reduce its expression. This hypothesis needs to be examined in a more systematic fashion. Whether *EPHB6* constitutes an example of such a gene remains to be determined.

## 12. Regulation of B-Lymphocyte Activity

B cells recognize antigens by means of their surface B-cell receptors (BCR), which consist of membrane immunoglobulin molecules (very small cytoplasmic region) and associated Igα/Igβ (CD79a/CD79b) heterodimers (large cytoplasmic region). The immunoglobulin-like tetramer binds the antigen, while the Igα/Igβ dimer transduces signals to the cell interior. Once stimulated with a cognate antigen, BCR directs the cell to activate and differentiate into antibody-generating plasma cells. Signal transduction begins with the phosphorylation of the so-called ITAM domains of the Igα/Igβ dimer. This creates a docking site for the recruitment and activation of the SYK kinase. The BCR/SYK complex activates several BCR-controlled signaling pathways: PLC-γ, PI3K, and MAPK [111].

The SYK kinase, in addition to BCR, mediates signal transduction downstream of a variety of transmembrane receptors, so its functioning is not restricted to the activation of B cells. In our experiments, the *SYK* gene was induced in A549 cells by A+N treatment. We detected it at the mRNA level in our RNA-Seq data [7] and at the protein level using Western blotting [17]. The upregulation of SYK mRNA was very strong (170-fold). In one individual study performed by others, the *SYK* gene was downregulated in cells with induced expression of p53 [81]. A review of transcriptomic data revealed the upregulation of *SYK* by p53 in only 12 reports (Table 1). We observed that A+N no longer induces accumulation of the SYK protein in p53-deficient cells, which definitely indicates that p53 is required for the stimulation of *SYK* expression, at least in A549 cells. Furthermore, we observed that SYK is upregulated in A549 cells not only by an unusual drug combination (A+N), but also by camptothecin, which is a precursor to the anticancer drugs topotecan and irinotecan. This protein may be upregulated in U-2 OS cells, but not in the melanoma A375 cell line, which also has a functioning p53 pathway [17]. Thus, the upregulation of SYK by p53 is cell-type specific. Considering the fact that the SYK kinase controls signaling from many receptors, we conclude that p53 may have a pleiotropic effect on various signaling pathways by activating the *SYK* gene.

*BLNK* constitutes another gene involved in the activation of B cells, which is positively regulated by p53 [57]. BLNK is an adapter protein in the BCR pathway and it is required for the coupling of the BCR receptor to the activation of the PLC-γ signaling pathway [112], which in turn, via several intermediate steps, activates NF-kappaB signaling. Without BLNK, B cells fail to proliferate in response to B-cell antigen receptor engagement [113]. BLNK mediates the inhibition of cytokinesis. The authors of the report concluded that in a pre-B leukemia model, BLNK acted as a p53 mediator in inhibiting cytokinesis, which prevents aneuploidy [57]. However, BLNK may also be involved in the positive regulation of Met oncoprotein signaling in non-small-cell lung cancer cells, which is counterintuitive for the product of a p53-target gene [114]. We found that BLNK is activated by A+N and camptothecin in a p53-dependent manner, and we identified the p53 response element in the BLNK promoter. There is a very strong synergy between actinomycin D and nutlin-3a in the activation of BLNK, which suggests that the p53 transcription factor must be very strongly activated in order to stimulate the expression of BLNK [7]. Twenty-three transcriptomic studies reported *BLNK* as a p53 target (Table 1). Thus, p53 has the ability to modify various signaling pathways by inducing expression of the BLNK adaptor protein.

## 13. Cytokines, Chemokines, and Other Secreted Signals

Cytokines affect the proliferation of various immune cells, whereas chemokines attract immune cells to their target locations. We observed that exposure of cells to A+N strongly (over 80-fold) stimulated the expression of the *IL7* gene coding for interleukin 7 (Table 1), which is a non-redundant growth factor for many hematopoietic cell lineages, especially for T and NK cells [115]. In p53-deficient cells, IL7 expression was strongly attenuated [53]. Transcriptomic studies have infrequently detected IL7 as a p53 target gene (Table 1), which suggests that activation of this gene is restricted to certain cell types or stress conditions.

*GDF15* constitutes another gene regulated by p53 [61] that codes for a secreted protein. It has been extensively studied due to the important role it plays in aging and the functioning of the immune system. Growth differentiation factor-15 is a distant member of the transforming growth factor TGF-β superfamily of cytokines. It is expressed in almost all tissues. Initially, this protein was discovered as an inhibitor of macrophage function, hence its other name MIC-1 (macrophage inhibitory cytokine-1). Subsequently, it was found to suppress the function of neutrophils, dendritic cells, NK cells, and T cells. Unexpectedly, GDF15 was found to be a ligand for a neuronal receptor involved in regulating body weight, prompting research into GDF-15 as a weight-loss promoter. It may also have anti-inflammatory properties by promoting an M2-like phenotype in macrophages (reviewed by Pence [116]). Despite all the data generated, the biological role of GDF-15 is still poorly defined. An interesting paper published recently indicated that the protein in question coordinates tolerance to inflammatory damage [117]. Some even call it the “disease tolerance cytokine” [118]. We found that the GDF15 gene is upregulated by A+N (10-fold). *GDF15* was identified as a p53 target in the overwhelming majority of high throughput studies (Table 1). Thus, p53 functions as an inducer of GDF-15 in many cell types and different treatment conditions. On the one hand, p53 stimulates the expression of immunostimulatory proteins, and, on the other, it promotes the expression of a protein which apparently inhibits the activity of many immune cells. To reconcile these observations, one explanation is that GDF-15 may function in negative feedback loops. Alternatively, it may promote tissue repair after infection is terminated. We will return to this discussion later herein.

An interesting gene activated by A+N is *EBI3* (Epstein–Barr virus induced 3). The gene is activated 10-fold by the A+N drug combination and has been found as a p53 target gene in 31 transcriptomic studies (Table 1). This gene codes for the subunits of two interleukins belonging to the interleukin-12 family. EBI3 forms interleukin-27 with the p28 protein, and interleukin-35 with the p35 subunit [119]. IL-27 regulates both pro-tumorigenic and anti-tumorigenic responses under various experimental conditions. Unlike IL-27, with its ambivalent role in immunity, IL-35 is mainly an immune suppressive cytokine [120]. Thus, the outcome of p53 stimulation of the *EBI3* expression is unknown.

The *CCDC3* gene encodes a secreted protein, coiled-coil domain containing 3, expressed in vascular-endothelial cells and in adipose tissue [121]. Exposure to A+N activates CCDC3, and it has been identified as a p53-activated gene in 20 transcriptomic studies (Table 1). It is a poorly-studied protein, although its immune-related function has been recognized. It represses TNF-α/NF-κB-induced pro-inflammatory response in vascular endothelial cells (Azad et al., 2014). Unexpectedly, the intracellular function of this protein has also recently been identified. This gene is directly stimulated by p53 in breast cancer cells and its protein apparently plays a role in the positive feedback loop regulating p53 activity, as it binds to the C-termini of p53 and MDM2, consequently stabilizing p53 in the nucleus and impairing MDM2 recruitment of p53 to the proteasome [58]. Thus, by activating *CCDC3*, p53 promotes its own activity. Furthermore, by means of the extracellular form of CCDC3, p53 regulates inflammation and lipid metabolism.

## 14. Genes for Bactericidal Proteins

The *LACC1* gene encodes a laccase 1 domain containing protein that exhibits oxidoreductase enzymatic activity. It is expressed in myeloid cells and is required for optimal fatty acid oxidation, mitochondrial ROS production, cytokine secretion, and bacterial clearance [122,123]. LACC1 deficiency has been found in humans and this has helped establish its role in immunity. Juvenile idiopathic arthritis is the most common chronic rheumatic disease in children. Homozygous LACC1 loss-of-expression mutations have been found in four such families [124]. Other genetic studies have demonstrated an association between LACC1 genetic variants and the risk of Crohn’s disease, leprosy, and inflammatory bowel disease ([122] and refs therein). Considering that most transcriptomic studies have identified *LACC1* as a p53-activated gene which is also highly upregulated by A+N (Table 1), it is surprising that it has not been identified as a p53-regulated gene in any individual report.

The *AZU1* gene encodes the heparin-binding protein (HBP) also known as azurocidin or the 37 kDa cationic antimicrobial protein (CAP37). It is located in neutrophil granules and is involved in a broad antimicrobial activity. HBP chemoattracts monocytes, induces leakage of blood vessels and the formation of edema. Moreover, HBP promotes the release of gamma interferon by macrophages (reviewed by Linder et al. [125]). The AZU1 gene is induced in cells exposed to A+N (it is not expressed in control cells), but it has not been frequently detected as a p53 target in transcriptomic studies (Table 1), perhaps because it is activated by p53 only under certain stress conditions. Nonetheless, the hypothesis that p53 contributes to the inflammatory response by promoting HPB production and release by non-neutrophil cells arriving at the site of inflammation is attractive and biologically plausible.

## 15. Negative Feedback Loops

Signaling pathways have various built-in feedback loops. These can be both positive, which help amplify the initial signal, and negative, which help terminate the signaling. In the case of the p53 signaling pathway, the main negative regulator is the aforementioned MDM2 protein, which is coded by a p53-activated gene. Thus, p53 activates the expression of its own inhibitor, which helps quench the signaling once its biological purpose is reached. MDM2 negatively regulates p53 by antagonizing the transcription activating domain thereof and by directing the entire protein to proteasomal degradation. Another negative regulator of p53 also coded by its target gene *PPM1D* is a phosphatase that removes activating phosphates from the p53 molecule [126]. Hence, it seems not to be unusual that an activator of a pathway also promotes the expression of a negative regulator.

The SOCS1 (suppressor of cytokine signaling 1) protein plays the role of a negative regulator in the signaling pathway that starts with interferon γ. The SOCS1 expression may be induced by cytokines, including various interleukins, interferons (type I and II), and TNFα. SOCS1 may inhibit signaling of almost all cytokines using Janus kinases (JAKs) for signal transduction, as it directly inhibits JAKs (except for JAK3). SOCS1 contains a “kinase inhibitory region”, which probably functions as a pseudo-substrate for JAKs and is essential for suppressing cytokine signals [127]. We have observed that when cells are exposed simultaneously to interferon α and A+N, the phosphorylation of STAT1 (on Tyr701) is lower compared with that in cells exposed to the interferon alone. This suggests that A+N treatment promoted the expression of the negative phosphorylation regulator STAT1. The A+N combination (or camptothecin) strongly induced the expression of the *SOCS1* gene at the mRNA and protein levels, and this activation was attenuated in p53-deficient cells [53]. A review of transcriptomic data revealed upregulation of SOCS1 by p53 in 12 reports, yet in 9 reports it was found to be thereby downregulated. This unusual situation and apparent contradiction may be explained by our unpublished observation indicating that the activation of p53 by nutlin-3a leads to downregulation of SOCS1 in some cells (e.g., U-2 OS) with high baseline SOCS1 gene expression. Therefore, SOCS1 may constitute a decent model for studying the double role of p53 in regulation of gene expression. The regulation of SOCS1 with the consequent influence on cytokine signaling is apparently cell-type specific and requires further, more detailed investigation.

SOCS1 inhibits cytokine signaling by preventing the phosphorylation of the STAT transcription factor by JAKs. A different mechanism is employed by the SMPDL3B protein (sphingomyelin phosphodiesterase, acid-like 3B). This protein is co-purified with toll-like receptors (TLRs), suggesting interaction of these proteins. Deficiency of SMPDL3B profoundly changes the cellular lipid composition and membrane fluidity. The physicochemical properties of the cell membrane significantly modulate signaling, starting with membrane receptors. Smpdl3b deficiency in mouse macrophages enhanced the response to TLRs, and Smpdl3b-deficient mice displayed an intensified inflammatory response. Thus, SMPDL3B is a negative regulator of TLR signaling [128]. Interestingly, the same authors demonstrated that in mouse macrophages this gene was stimulated by interferon γ and various TLR ligands, again exemplifying the principle that an activator of a signaling pathway promotes the expression of an inhibitor as part of a negative feedback loop. Overall, SMPDL3B is not a well-studied protein. The gene is strongly activated by the A+N combination, and in transcriptomic analyses it was found to be upregulated by p53 in 16 reports (Table 1). Hence, not only does p53 stimulate the expression of some TLRs, but it may also promote the expression of their negative regulators.

Another element of the negative feedback loop in interferon signaling is the aforementioned SLFN5 protein, a transcriptional co-repressor of STAT1. Type I interferon treatment upregulates the expression of SLFN5, which interacts with STAT1 inhibiting the activation of interferon-stimulated genes. SLFN5 is both an IFN-stimulated response gene and a repressor of IFN-activated gene transcription [129]. Its activation by A+N and the transcriptomic data (Table 1) suggest that *SLFN5* constitutes a p53-target gene involved in the negative regulation of the interferon signaling pathway (Figure 3).

The SPRY1 protein is a negative feedback inhibitor of growth factor receptor signaling, including immune-related receptors. It modulates the signaling from the T-cell receptor [130,131]. A+N upregulated the *SPRY1* expression 14-fold, and transcriptomic studies have detected *SPRY1* as a p53 target in 20 reports (Table 1). Therefore, p53 may modulate signaling by inducing expression of SPRY1, starting with the TCR but also including other growth-promoting cell membrane receptors.

The PIK3IP1 protein is involved in the negative regulation of phosphatidylinositol 3-kinase (PI3K) activity and, consequently, the negative regulation of the phosphatidylinositol 3-kinase pathway—the major growth-promoting signaling system. This pathway is subject to a significant negative regulation at the PIP_3_ second messenger level, which is removed by various phosphatases including PTEN and SHIP1. PIK3IP1 negatively regulates the pathway, acting at the level of PI3K activity itself. PIK3IP1 mRNA is highly expressed in T and B lymphocytes. Experiments revealed that PIK3IP1 constitutes a novel regulator of PI3K activity in lymphocytes (reviewed by Murter and Kane, [132]). A recent report revealed that downregulation of Pik3ip1 in mouse T cells caused a major metabolic shift from oxidative phosphorylation toward aerobic glycolysis, leading to their overactivation and aggressive autoimmune disease. This is a previously unrecognized role of Pik3ip1 in metabolic regulation, substantially affecting inflammation and autoimmunity [133]. In addition, *Pik3ip1*-deficient mice exhibited an enhanced T-cell response and a marked increase in antitumor immunity upon immunization with a neoantigen [134]. Considering the fact that the *PIK3IP1* gene is strongly upregulated by A+N and has frequently been detected as a p53-regulated gene in transcriptomic studies (Table 1), it may be concluded that through positive regulation thereof, p53 modulates PI3K activity, metabolism, and T-cell activation. The hypothesis that p53 positively regulates the expression of *PIK3IP1* is supported by its positive regulation by A+N and the results of transcriptomic studies. Coronel et al. [135] also reported that p53 directly activates the RFX7 transcription factor gene, which in turn directly activates the transcription of the *PIK3IP1* gene. Thus, *PIK3IP1* is an example of a subset of the genes regulated indirectly by p53.

## 16. Immunological Tolerance

The balance between activating and inhibitory signals delivered to effector immune cells requires precise regulation. Suboptimal activity may lead to higher risk of infections and cancer, while excessive activity may lead to autoimmune diseases. In this section, we investigate the genes regulated by p53 that may promote immunological tolerance. It is sometimes difficult to distinguish between parts of the negative feedback loop and elements promoting immune tolerance, hence the placement of a gene in this or the previous section is somewhat arbitrary.

In order to elicit protective immunity or to inhibit an overactive immune system, the immune response needs to be strictly controlled by the proteins of the B7 family. The B7 family contains co-stimulatory and co-inhibitory molecules. As mentioned above, T-cell activation depends on two signals: signal 1, (antigen recognition), where peptides presented by the MHC class I complex are identified by T-cell receptors (TCRs); and signal 2, or co-stimulation, involving the combination of co-regulators such as B7 proteins, consisting of co-stimulatory and co-inhibitory molecules expressed on antigen-presenting cells (APCs). Signals from the B7 family members are critical for preserving a balance between immune potency and autoimmunity suppression. The famous molecule PD-L1, which potently inhibits T cells and is involved in target anticancer immunotherapy, belongs to the B7 family [136]. This family also contains an Ig-like domain-containing receptor 2 encoded by the *ILDR2* gene. This protein demonstrates a robust T-cell inhibitory activity. It is highly expressed in the testes and moderately in the brain. Its expression is elevated in inflamed tissues in patients with ulcerative colitis and Crohn’s disease, and also during differentiation of human monocytes into macrophages [137]. Additional studies have demonstrated that this poorly-studied protein has potential for use in the treatment of autoimmune diseases [138]. The immunosuppressive activity of ILDR2 may be blocked by a recently-developed antibody with cancer immunotherapy potential [139]. Transcriptomic studies have demonstrated that *ILDR2* was found to be activated by p53 in 14 reports, and A+N activated it more than 700-fold (Table 1). Moreover, our unpublished results indicate that this gene shows high baseline expression in U-2 OS cells (frequently used in transcriptomics studies), where it is not further increased by A+N, but it is strongly upregulated thereby in two other cell lines derived from lung cancer or melanoma. Furthermore, the promoter region of *ILDR2* contains a p53 binding site, which, according to the ChIP-Seq data, is not occupied by wild-type p53 but by a mutant that promotes cooperative binding of p53 monomers [56]. Thus, apparently “regular” activation of p53 seems not to be sufficient to stimulate the expression of *ILDR2*. Assuming that p53 actually activates the expression of this gene, it appears very puzzling why the tumor suppressor promotes the expression of a robust T- cell inhibitor. We would expect a tumor suppressor to promote the destruction of cancer cells by the immune system. One explanation is that the stress evoked by A+N primes the cells to other types of death than destruction by cytotoxic T cells, but this hypothesis awaits testing.

Another gene involved in immune tolerance, *INPP5D*, codes for a phosphatase that removes a phosphate group from the second messenger—PIP_3_ of the phosphatidylinositol 3-kinase pathway mentioned above. Thus, INPP5D (also known as SHIP1) constitutes another negative regulator of the pathway in question. INPP5D plays a role in the negative regulation of TLR3-induced production of type I interferons (Figure 3) [140], yet it also functions in other signaling systems that regulate the activity of T cells, B cells and NK cells. INPP5D deficiency is associated with autoimmunity (reviewed by Pauls and Marshall, [141]). *INPP5D* has been identified as a p53-regulated gene [63]. Thus, it appears that p53 has the ability to inhibit the phosphatidylinositol 3-kinase pathway by upregulating the expression of *INPP5D*. This hypothesis is supported by the observation that most transcriptomic studies have demonstrated that p53 positively regulates its expression and it is strongly activated by the A+N combination (Table 1). Unexpectedly, transcription of this gene in A549 cells exposed to A+N begins with an internal promoter that drives the production of mRNA starting from exon 8 (transcriptomic data published by Łasut-Szyszka et al. [7]). When this mRNA is translated, it produces a protein lacking the SH2 domain at the amino terminus. This domain participates in binding to cell membrane receptors, e.g., those present on immune cells (reviewed by Pauls and Marshall, [141]). Thus, it is plausible that INPP5D induced by A+N fails to inhibit phosphatidylinositol 3-kinase signaling in immune cells. These observations will be extended by further research. Our unpublished transcriptomic data demonstrate that a similar transcript is generated in A549 cells exposed to camptothecin and to nutlin-3a acting alone, as well as in other cell types.

## 17. Concluding Remarks

The best-known and -documented biological role of p53 is protection against neoplastic disease. This conclusion is supported by the frequent presence of *TP53* mutations in human cancers and very high cancer risk in carriers of its germline mutations [142]. However, the molecular mechanisms of protection are not yet deeply understood [1]. The immune system stimulation may constitute an important part of the antineoplastic role of p53. The described activity of p53 may also boost protection against pathogens, including viruses. However, the observation that p53 strongly activates the expression of some immunosuppressive genes is not consistent with the immunostimulatory role of p53. Does p53 really suppress our immune system? (This seems highly unlikely for a tumor suppressor.) Is p53 involved in negative feedback loops in immune-related signaling pathways? Is p53 such a versatile transcription factor in some stress conditions that the activation of immunity genes is merely a side-effect? Or maybe we just do not comprehend the intricate connections between p53 and various components of the immune system. In order to answer these questions and others concerning p53, more work based on a better in vivo experimental model is needed.

## Figures and Tables

**Table 1:** The selected genes associated with immunity that are strongly activated by treatment with actinomycin D and nutlin-3a (A+N). The third column shows the number of transcriptomic studies that identified the gene as a p53 target, from the set of 57 reports reviewed by Fischer et al. [8].

Gene Name	Fold-Change A+N [7]	Number of Transcriptomic Studies Identifying the Gene as p53 Target [8]	p53 Target in Individual Reports
AZU1	inf	6	No reports found
BLNK	1300	23	[57]
CASP1	1500 *	16	[54]
CCDC3	12.8	20	[58]
CD82	inf	45	[59]
CEACAM1	149.1	31	[60]
DOCK8	14.4	26	No reports found
EBI3	10.1	31	No reports found
EFNB1	26.0	46	No reports found
EPHB6	inf	9	No reports found
ERAP2	5.4	30	No reports found
GDF15	9.6	53	[61]
GLIPR2	7.9	29	No reports
IFI16	17.3	8	[62]
IL7	85 *	10	[53]
ILDR2	724.1	14	No reports found
INPP5D	71.5	50	[63]
IRF5	6.7	17	[64]
IRF7	4.1	6	[48]
IRF9	4.0	4	[65]
ISG15	45.3	30	[66]
KCNK6	7.5	27	No reports found
LACC1	10.8	46	No reports found
NLRP1	500 *	36	[53]
PSMB8	5.9	10	No reports found
PYHIN1	364.6	6	No reports found
SH2D2A	30.1	20	No reports found
SLFN5	8.8	39	No reports found
SMPDL3B	27.5	16	No reports found
SOCS1	23.9	12	[53]
SPRY1	13.9	20	No reports found
STING1	10.6	27	[53]
SYK	168.9	12	[17]
TAP1	9.8	50	[67]
TLR3	28.4	28	[68]
TREM2	inf	21	[17]
TRIM22	59.7	47	[69]
TYROBP	inf	No data	[17]
ULBP2	14.0	23	[70,71]

inf—infinity, when expression in control cells is 0. * Based on the reports by Krześniak et al. [53].

## Data Availability

No new data were created or analyzed in this study. Data sharing is not applicable to this article.
